# Ventilatory Chemosensory Drive Is Blunted in the *mdx* Mouse Model of Duchenne Muscular Dystrophy (DMD)

**DOI:** 10.1371/journal.pone.0069567

**Published:** 2013-07-29

**Authors:** Matias Mosqueira, Santhosh M. Baby, Sukhamay Lahiri, Tejvir S. Khurana

**Affiliations:** 1 Department of Physiology, University of Pennsylvania School of Medicine, Philadelphia, Pennsylvania, United States of America; 2 Pennsylvania Muscle Institute, University of Pennsylvania School of Medicine, Philadelphia, Pennsylvania, United States of America; 3 Medical Biophysics Unit, Institute of Physiology and Pathophysiology, Heidelberg University, Heidelberg, Germany; University of Minnesota Medical School, United States of America

## Abstract

Duchenne Muscular Dystrophy (DMD) is caused by mutations in the DMD gene resulting in an absence of dystrophin in neurons and muscle. Respiratory failure is the most common cause of mortality and previous studies have largely concentrated on diaphragmatic muscle necrosis and respiratory failure component. Here, we investigated the integrity of respiratory control mechanisms in the *mdx* mouse model of DMD. Whole body plethysmograph in parallel with phrenic nerve activity recordings revealed a lower respiratory rate and minute ventilation during normoxia and a blunting of the hypoxic ventilatory reflex in response to mild levels of hypoxia together with a poor performance on a hypoxic stress test in *mdx* mice. Arterial blood gas analysis revealed low PaO_2_ and pH and high PaCO_2_ in *mdx* mice. To investigate chemosensory respiratory drive, we analyzed the carotid body by molecular and functional means. Dystrophin mRNA and protein was expressed in normal mice carotid bodies however, they are absent in *mdx* mice. Functional analysis revealed abnormalities in Dejours test and the early component of the hypercapnic ventilatory reflex in *mdx* mice. Together, these results demonstrate a malfunction in the peripheral chemosensory drive that would be predicted to contribute to the respiratory failure in *mdx* mice. These data suggest that investigating and monitoring peripheral chemosensory drive function may be useful for improving the management of DMD patients with respiratory failure.

## Introduction

DMD is the most common X-linked fatal neuromuscular disorder. The disease affects males from early childhood and death usually occurs in the second to third decade of life due to ventilatory and cardiac failure [1], [2], [3], [4], [5], [6]. The disease is caused by mutations in the DMD gene resulting in an absence of dystrophin in muscle and neurons. While muscular weakness and wasting are the most well recognized signs and symptoms of DMD, some but not all patients also present with neurological manifestations exemplified by mental retardation and abnormalities in retinal function [7], [8]. Dystrophin is a 427 KDa protein, located sub- sarcolemmally and forms a critical part of the dystrophin- glycoprotein complex. In muscle, the absence of dystrophin renders the sarcolemma susceptible to damage and degeneration by mechanical forces during repeated cycles of muscle contraction and relaxation [9], [10], [11]. The progressive muscle damage resulting in necrosis and fibrosis has been extensively studied in DMD patients. Histological analysis of limb muscles from DMD patients reveal overexpression of TGF-β1, connective tissue growth factor, proliferation of heparin-sulphate proteoglycans, collagen type III, adipose tissue and infiltration of immune cells [12], [13], [14], [15], [16], [17]. The consequence of the structural tissue damage in DMD patients is an impairment of respiratory function, which has been described as a restrictive ventilatory syndrome [18], [19], [20], [21], [22]. DMD patients show a positive correlation between the rates of decrease in vital capacity (VC) and forced expiratory volume in 1 second (FEV_1_) and a decrease in maximal inspiratory pressure as the disease progresses [23]. Other factors, such as an alteration of the elastic properties of elastin and collagen fibers, a decrease in surfactant activity and fibrosis reduces the ventilatory system compliance [24], [25], [26], [27]. The decrease of functional residual capacity and maximal inspiratory effort is correlated with an increase of the diaphragm thickness in DMD patients due to increased connective tissue [28]. Muscle damage in the diaphragm and other respiratory muscles leads to a reduced trans-diaphragmatic pressure, peak inspiratory flow during forced VC and tidal volume, which in turn leads to a chronic condition of hypercapnia and hypoxemia and eventually respiratory failure [3], [4], [5], [29], [30], [31].

The carotid body (CB) is the primary peripheral chemoreceptor that responds to hypoxemia, hypercapnia and acidosis stimuli. These stimuli increase the minute ventilation in order to reestablish the arterial oxygen and/ or carbon dioxide pressure and/ or pH via CB efferents [32], [33]. In the CB, the cellular response to hypoxia is mediated by the hypoxia inducible factor (HIF) [34], [35], [36] pathway as well s transcription factors including AP-1, NFκB and TP53 [37]. Both, systemic and cellular responses to hypoxia are considered to be defensive responses; however, the chronic exposure to hypoxia can result in various pathological situations, including *cor pulmonale* [38].

The murine animal model (*mdx*) of DMD has extensively been used to understand the pathophysiology of the disease. In particular, the *mdx* mouse diaphragm faithfully reproduces many patho-physiological and histological features seen in human DMD patients [1], [39], [40], [41], [42]. It has been described that the dystrophic diaphragm shows a significant contractile fatigue and has a reduced ability to precisely and consistently generate force following both nerve and muscle stimulation at 35 Hz [43]. Several ventilatory studies have been performed in *mdx* mice [44], [45], [46], [47], [48], [49], [50] that in common document the respiratory involvement, however, yielding some inconsistencies in terms of specific features of their responses to hypercapnic [47], [50], normoxic and hypoxic stimuli [46], [48], [49]. Despite being well studied, lacunae exist regarding the peripheral chemosensory drive in dystrophin-deficiency and there is a lack of consensus about the integrity of basic ventilatory reflexes in *mdx* mice. To address these issues we studied ventilatory parameters using a non-invasive whole body plethysmograph (WBP), as well as performed examination of the phrenic nerve activity and the arterial blood gases from adult *mdx* mice. To determine the potential role of dystrophin in the peripheral chemosensory reflexes, we studied the expression of dystrophin mRNA and protein in the CB peripheral arterial chemoreceptor, as well as performed functional assessment of CB chemosensory drive in normal and *mdx* mice.

## Materials and Methods

### Animals

Adult male normal (C57BL/10ScSn) and *mdx* (C57BL/10ScSn-Dmd*^mdx^*/J) mice obtained from the Jackson Laboratory (Jackson Laboratory, Bar Harbor, ME) were kept in the animal facility at the University of Pennsylvania School of Medicine until 6–7 month of age. All animal experiments were performed according to U.S. laws and were approved by the Institutional Animal Care and Use Committee at the University of Pennsylvania, School of Medicine, and all effort were made to minimize suffering.

### Measurement of Respiratory parameters via WBP

To measure respiratory parameters as well as HVR and HCVR, we custom-built a non-invasive WBP chamber (radius 2.5 cm; height 10.0 cm) with gas administration ports and a polyethylene base, with a final volume of 150 mL (Figure S1A). A MLT1L pneumotachometer (AD Instruments Inc., Colorado Springs, CO) was connected to the outlet port and the difference of pressure integrated using a ML140 Spirometer module (AD Instruments). The analog signal was converted to digital using Power Lab 8/SP (AD Instruments), filtered at 10 Hz and acquired at 400 B/s with the Chart 4.2.4 data acquisition system (AD Instruments) and stored in a PC. Calibration was performed according to the manufacturer's manual.

### HVR and HCVR protocols

Unanesthetized freely-moving normal and *mdx* mice age 6 to 7 months were allowed to acclimatize to the WBP chamber for 30–60 min/ day for 4 days while flushing with fraction of inspired oxygen (FiO_2_) 21% at 0.5 L/ min before the actual experiments. Required gas mixtures were obtained using a commercial gas mixer (Pegas-400 MF Gas Mixer, Columbus Instruments, Columbus, OH, USA) set at 1 L/ min combined with a manual flow meter to regulate the flow into the chamber at a final flow rate of 0.5 L/ min. To determine the HVR, acclimatized normal and *mdx* mice (n = 5 each), were subsequently exposed to FiO_2_ of 100, 21, 18, 15, 12, 10 and 8% O_2_- balanced in N_2_ each interspaced by 10 minutes of normoxia until a steady response was obtained. To determine the HCVR, the animals were subsequently exposed to fractions of inspired CO_2_ (FiCO_2_) e.g. 5% CO_2_-balanced with air, 5% CO_2_-balanced with 95% O_2_, 10% CO_2_-balanced with air and 10% CO_2_-balanced with 90% O_2_ until a steady response was recorded. The different hypercapnic challenges were interspaced by 10 minutes of normoxia. Normalized minute ventilation (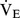, µL/ s/ g) measured from normal and *mdx* mice was calculated from the respiratory rate (*f*_R_, Hz) multiplied by tidal volume normalized to body weight (V_T_, µL/ g). The analysis was performed from three different measurements with at least 20 s of steady breathing without behavioral and/ or physical activity of the animals. To avoid interference of thoracic movements at V_T_ values during inspiration and expiration, a correction factor was multiplied to normalized V_T_. This correction factor was obtained from the ratio between the normalized V_T_ of the nose plethysmograph and the normalized V_T_ of the WBP from five different normal mice. The detailed protocol description is available at TREAT-NMD website: SOP- Respiratory System Evaluation, DMD_M.2.2.002: http://www.treat-nmd.eu/downloads/file/sops/dmd/MDX/DMD_M.2.2.002.pdf

### Hypoxic stress test

To determine the resistance to severe hypoxia, anesthetized age matched normal and *mdx* mice (n = 14) were exposed to FiO_2_ 8% (hypoxia) in the WBP chamber, as described above. Hyperventilation and ventilatory disturbances during FiO_2_ 8% were monitored carefully to avoid fatality. The time (in seconds) between the beginning of tachypnea and the first apneic pause after being exposed to FiO_2_ 8% was measured.

### Phrenic nerve activity

Age matched normal and *mdx* mice were anesthetized with ketamine (80 mg/Kg) and xylazine (20 mg/Kg), followed by an additional dose of ketamine when required to maintain surgical level of anesthesia. Phrenic nerve activity was monitored as an index of central respiratory neuronal output. The phrenic nerve was isolated unilaterally at the level of the C5 and C6 spinal segments, placed on a bipolar platinum electrode and lifted on mineral oil. The neural discharge was pre-amplified using an AC Preamplifier Model P-15 (Grass Instruments, Quincy, Massachusetts, USA), filtered (100 Hz – 3 KHz), the analog signal was converted to digital using a PowerLab (AD Instruments), acquired at 1 KB/s using Chart 4.2.4 (AD Instruments). To determine the hypoxic response on the phrenic nerve activity, animals (n = 4 each) were exposed three times to each FiO_2_ 100, 21, 8, 4 and 0% O_2_-balanced in N_2_, until a steady response was recorded. Care was taken to avoid death by anoxia. The frequency of phrenic nerve discharge and each burst of the phrenic nerve activity were analyzed from three different responses of the same FiO_2_ challenge.

### Arterial blood gas analysis and hematocrit

The carotid artery was cannulated with heparin-coated PE-50 tubing in anesthetized animals for arterial blood sampling. Two arterial blood samples were collected and immediately injected into an ABL700 Radiometer (Radiometer America, USA) to measure oxygen and carbon dioxide partial gas pressure (PO_2_ and PCO_2_) and pH. The hydrogen carbonate (HCO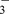) was calculated by an ABL700 Radiometer from PaCO_2_ values. The hematocrit was calculated from the percentage of red blood cells in the total blood sample after centrifugation (Micro-capillary centrifuges model MB, International equipment Co. Boston, MA).

### RNA isolation and qPCR

Total RNA was extracted with TRIzol reagent (Invitrogen) from *tibialis anterior* muscle and pooled CBs from age-matched normal and *mdx* mice and purified using the RNeasy kit (Qiagen, Valencia, CA) following the manufacturer's instructions. Five µg of total RNA was reverse transcribed using random hexamers (Invitrogen) and the SuperScript™FirstStrand enzyme (Invitrogen). The mouse dystrophin TaqMan assay Mm00464475_m1 from Applied Biosystems (Applied Biosystems, Foster City, CA) was used to determine dystrophin mRNA expression. cDNA corresponding to 25 ng of total RNA was amplified in 20 µl of reaction mixture-containing 1 µl of primer mix and 10 µl of 2× TaqMan universal PCR master mix (Applied Biosystems). The amplification was performed in a 7900HT Sequence Detection System (Applied Biosystem).

### Immunoblotting

Total protein was extracted from the CBs of normal and *mdx* mice with TNEC (50 mM Tris-HCl, pH 8.0; 150 mM NaCl; 1% NP40; 2 mM EDTA) buffer containing complete protease inhibitor cocktail (Roche, Basel, Switzerland). Protein concentration was determined using the Bradford Assay (Bio-Rad, Hercules, CA). For dystrophin immunoblotting, 50–75 µg of total protein was resolved on 3–8% Tris-Acetate gradient gels (NuPage; Invitrogen) and electro-transferred onto poly-vinylidene difluoride (PVDF) membranes (Immobilon P, Millipore, Billerica, MA). After blocking with 5% nonfat-milk in wash buffer (0.05 M Tris, 0.15 M NaCl, 0.05% Igepal CA630 and 0.1% BSA), blots were incubated overnight with dystrophin antibody (1∶500, Vector Laboratories, Burlingame CA) at 4°C. Blots were washed and incubated with either goat anti-rabbit or anti-mouse HRP-conjugated secondary antibodies at 1∶20,000 (Jackson ImmunoResearch) dilutions to detect protein bands using an enhanced chemiluminescence kit (Pierce). Membranes were re-probed with anti-mouse α-tubulin (1∶6000; Sigma) to confirm equal loading (data not shown).

### Dejours test and analysis of the HCVR fast component

Age-matched normal and *mdx* mice were anesthetized with a ketamine- xylazine mix as described. Using a custom made mouse mask (adapted to the mouse's snout; Figure S1B) attached to a pneumotachometer, signal acquisition and A/D conversion was performed as mentioned above. Four animals per group were breathing FiO_2_ 21% for 3 min and quickly switched to FiO_2_ 100% for 60 s.

A similar procedure was performed to test the HCVR fast component. A different set of 4 anesthetized animals per group were breathing FiO_2_ 21% for 3 minutes and then were quickly switched to either air-balanced hypercapnia (FiCO_2_ 5%- FiO_2_ 21%) or O_2_-balanced hypercapnia (FiCO_2_ 5%- FiO_2_ 95%) for 60 s. The analysis of *f*_R_ and V_T_ was performed in the first 10 s of either air-balanced hypercapnia or O_2_-balanced hypercapnia exposure. The percentage of ventilatory change from preceding normoxia exposure to either hyperoxia (Dejours test), air-balanced hypercapnia or O_2_-balanced hypercapnia was calculated.

### Statistical Analysis

All data were analyzed using GraphPad Prism 5.0 software (GraphPad Inc., La Jolla, CA). Respiratory and phrenic nerve activity differences were analyzed by a non-parametric ANOVA and Dunn post-test. The frequency of ventilation and discharge of the phrenic nerve was analyzed using a sigmoidal dose-response curve for variable slope. The arterial blood gases, Dejours test and the fast component of hypercapnia responsiveness data were analyzed using a non-parametric t-test. Values were considered statistically significant if p<0.05. The tests as described above were repeated three times for each animal. All results are shown as the mean ± SEM.

## Results

### Blunted HVR to mild hypoxia in *mdx* mice

**Figure 1:**
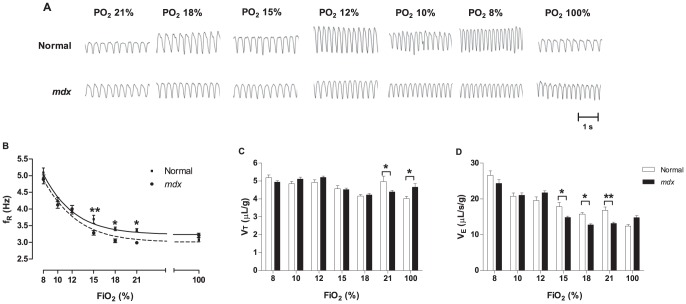
HVR of normal and *mdx* mice exposed to different FiO_2_ levels. A) Representative inspiratory and expiratory flows obtained from normal (top) and *mdx* mice (bottom) exposed to FiO_2_ 21, 18, 15, 12, 10, 8 and 100%. Each animal was challenged three times with each FiO_2_. The scale for tidal volume (V_T_, µL) and time (s) are shown at bottom right. B) Quantification of respiratory frequency (*f*_R_, Hz), C) tidal volume normalized by body weight (µL/g) and D) minute ventilation (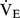) normalized by body weight (µL/s/g) from normal (empty bars) and *mdx* (filled bars) mice at each FiO_2_ levels tested. Mean ± SEM; * p<0.05; ** p<0.01, n = 5.

To study hypoxia ventilatory response (HVR) and hypercapnia ventilatory response (HCVR), normal and *mdx* mice were acclimated to a custom-built WBP chamber using room air (equivalent of a FiO_2_ of 21%). Mice were then challenged with different FiO_2_ and FiCO_2_ levels and *f*_R_, V_T_ and 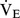 values obtained HVR was tested in normal and *mdx* mice using different FiO_2_ values (Figure 1A). *Mdx* mice showed a significantly lower *f*_R_ reduced V_T_ and 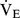 when exposed to room air (Fig. 1A, 1B, 1C and Table S1). A significant reduction in the *f*_R_ was observed during mild hypoxic challenges (FiO_2_ 18 and 15%), but not during moderate (FiO_2_ 10 and 12%) and severe hypoxic challenges (FiO_2_ 8%; Figure 1B). Although there were no significant changes in normalized V_T_ during hypoxic challenges in *mdx* relative to normal mice, *mdx* mice showed significantly lower normalized V_T_ values at FiO_2_ 21% and higher V_T_ values at FiO_2_ 100% (Figure 1C). 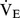 was significantly different at the same FiO_2_ levels as noted for *f*_R_ (Figure 1D).

**Figure 2:**
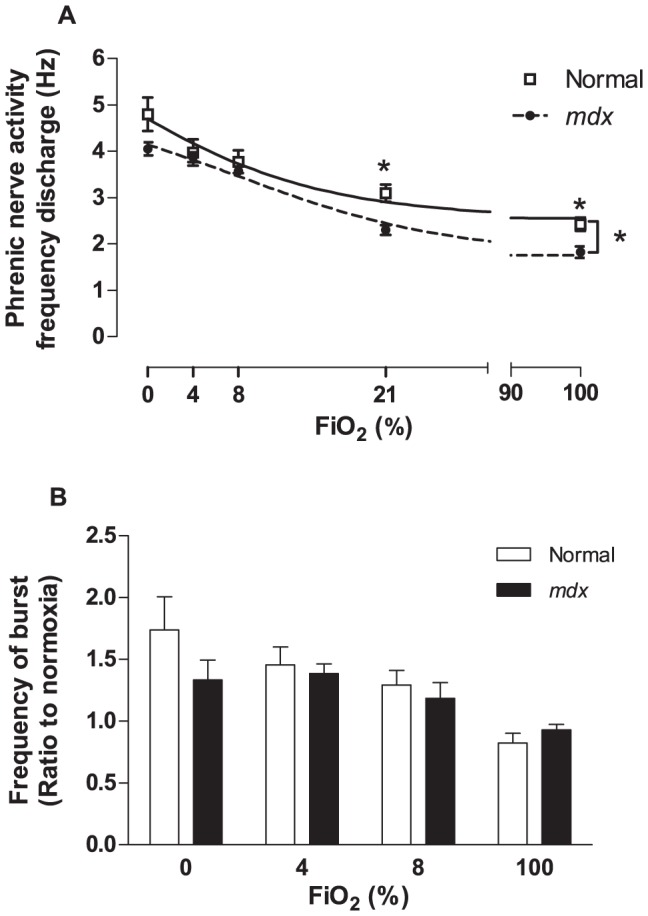
Phrenic nerve activity of anesthetized normal and *mdx* mice in response to hypoxia and hyperoxia challenges. A) Analysis of phrenic nerve activity frequency of discharge (*f*_x_, Hz) from normal (square, continuous line) and *mdx* mice (filled circle, broken line) in response to FiO_2_ 0, 4, 8, 21 and 100%. Each animal was challenged three times with each FiO_2_. B) Analysis of the average of each phrenic nerve burst during the response to FiO_2_ 0, 4, 8 and 100%. The data was normalized by the normoxic response activity (FiO_2_ 21%). Mean ± SEM; * p<0.05, n = 5.

To determine whether the blunted response to hypoxia was due to a lower phrenic nerve activity (*f*_x_) input to the main respiratory muscle, we extended the hypoxic challenge in anesthetized animals to FiO_2_ 0, 4 and 8% in addition to FiO_2_ 21 and 100% and recorded *f*_x_. Analysis showed a significant reduction of *f*_x_ in *mdx* mice during normoxia (FiO_2_ 21%) and at FiO_2_ 100% compared to normal mice (Figure 2A), similar to the *f*_R_ response pattern described above using WBP. These results suggest a blunted ventilatory response in *mdx* mice compared to normal mice (Table S2). As previously described in other mice strains [51], [52], the difference between the normoxic *f*_x_ and normoxic *f*_R_ results observed here in both normal and *mdx* mice was due to the effect of anesthesia. We also analyzed in both groups the phrenic nerve burst frequency, which showed no significant change at different levels of FiO_2_ (Figure 2C), suggesting that the phrenic nerve activity and the central generator of ventilation in the medulla were intact.

**Figure 3:**
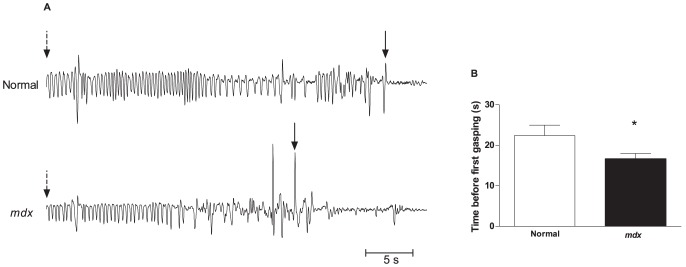
Time between the beginning of tachypnea and the first apneic pause during severe hypoxic challenge. A) Normal and *mdx* mice were exposed from normoxia to severe hypoxia (FiO_2_ 8%) and the time (seconds) between the hypoxia-induced tachypnea response (arrows) and apneic pause (arrow heads) was calculated. Each animal was challenged twice. B) Quantification of the time to first apneic pause. Mean ± SEM; * p<0.05, n = 14.

The volitional requirements of clinically useful tests of pulmonary functions (e.g. FVC and FEV_1_) precluded measurements in mice; hence we designed a hypoxic stress test to estimate ventilatory capacity and integrity of respiratory drive in mice. We observed that a quick change from FiO_2_ 21% to FiO_2_ 8% induced apneic pauses in both normal and *mdx* mice after a few seconds of hyperventilation (Figure 3A). Compared to normal mice the lapsed time between the beginning of hyperventilation induced by severe hypoxic challenge and the first apneic pause was significantly reduced in *mdx* mice (22.40±9.67 *vs.* 16.67±4.90 s; Figure 3B). This result suggests impairment in the ventilatory response mediated either by reduced muscle function in response to acute intense hypoxia induced activity in *mdx* mice exposed to severe hypoxic conditions and/or a reduced or altered respiratory drive.

### Normal HCVR in *mdx* mice

In order to separate the CB chemosensory hypercapnic response from the central (brainstem) hypercapnic response [53], we used two different types of hypercapnic mixes: CO_2_ balanced in air [to induce both the peripheral (CB) and central chemosensory response] and CO_2_ balanced in O_2_ (to induce the central chemosensory response only and make the CB chemosensory response feeble or absent). The analysis was performed after ca. 60 s of the hypercapnic stimulus, once the ventilation showed a stable response without interference by the animal's behavior. Compared to the normoxic response, *mdx* mice presented a significantly higher *f*_R_ and normalized V_T_ response at any hypercapnic level (Table S3). However, compared to normal mice, *mdx* mice did not show any significant change in the magnitude of change in response to hypercapnia in *f*_R_, V_T_ and hence in 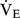 (Figure S2). These results suggested that HCVR in *mdx* mice at 6–7 months of age did not differ from normal mice.

### Altered arterial blood gases and hematocrit levels in *mdx*

**Figure 4:**
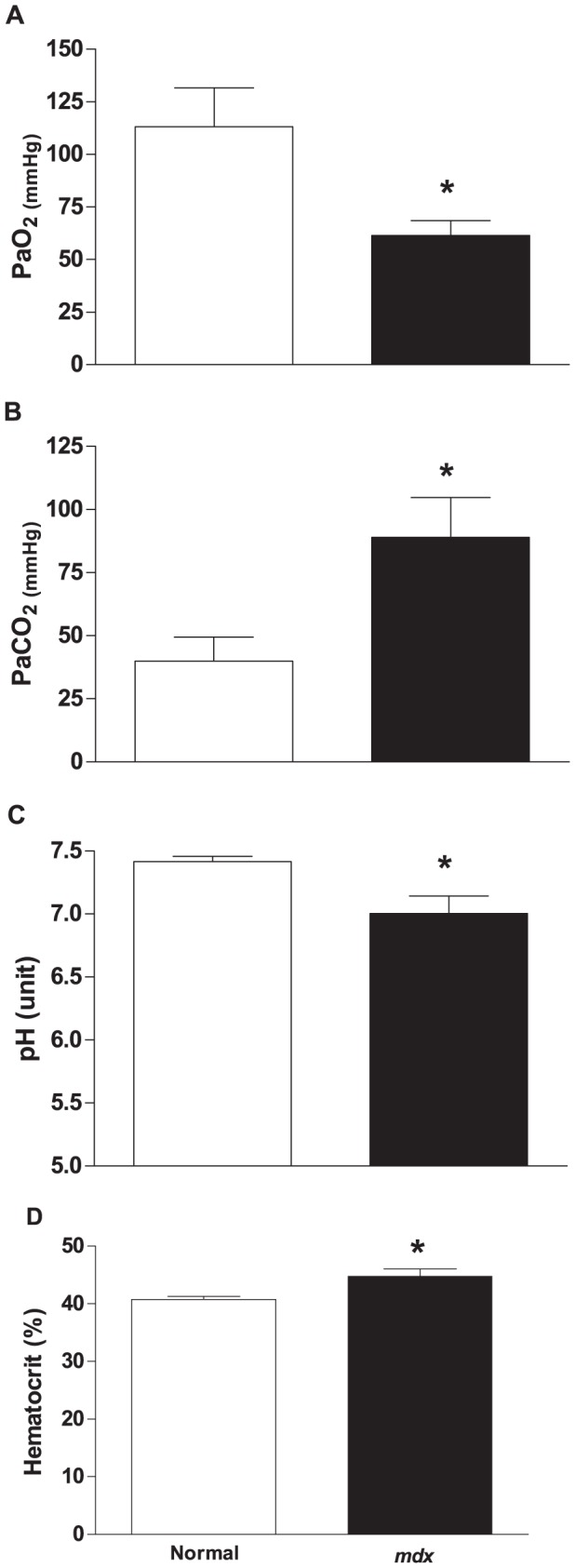
Arterial blood gases and hematocrit values obtained from normal and *mdx* mice during normoxic ventilation. Three arterial blood samples from each of five anesthetized normal (empty bars) and *mdx* (filled bars) mice were taken to measure A) PaO_2_, B) PaCO_2_, C) pH, and D) hematocrit. Mean ± SEM; * p<0.05.

Having established that *mdx* mice had lower *f*_R_ and V_T_ in normoxia and a blunted HVR to mild hypoxia, we asked if these changes in respiratory parameters would lead to alterations in arterial blood gasses during normoxic ventilation. Indeed, compared to normal mice, the *mdx* mice presented reduction of PaO_2_ (113.2±18.0 *vs.* 61.56±7.04 mmHg; Figure 4A), increased PaCO_2_ (39.98±9.56 *vs.* 89.02±15.70 mmHg, Figure 4B). Along with the reduced PaO_2_ and increased PaCO_2_ the arterial blood of the *mdx* mice had decreased pH (7.42±0.04 *vs.* 7.00±0.14; Figure 4C) compared to normal mice. There was a tendency towards lower levels of HCO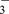 in *mdx* arterial blood (24.76±4.13 *vs.* 21.18±4.56 mmHg); however, this was not significant. The hematocrit of arterial blood (Figure 4D) was significant higher in *mdx* mice relative to normal mice (40.75±0.50 *vs.* 44.75±1.32).

### Absence of dystrophin in the *mdx* carotid body

**Figure 5:**
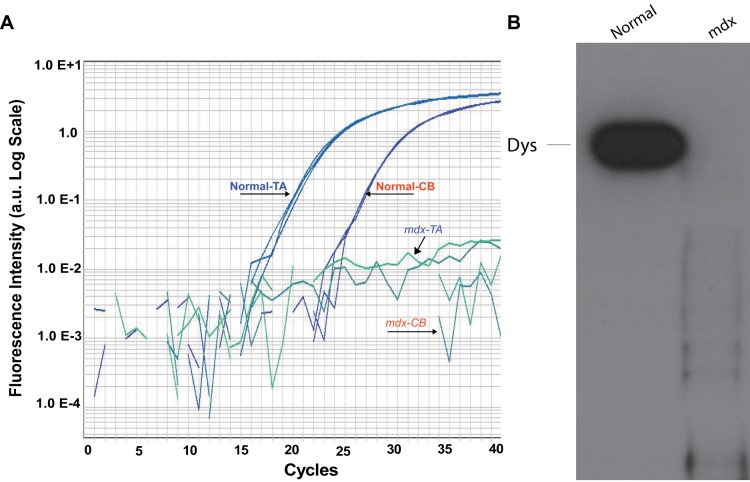
Expression of dystrophin in the CB from normal and *mdx* mice. A) Dystrophin mRNA expression level in the CB from normal mice is similar to that in *tibialis anterior*, whereas dystrophin mRNA was not detected in the two tissues from *mdx* mice. The delta Ct values obtained were TA 18. 85±0.31 and CB 25.51±0.07 (mean ± SD). Dystrophic TA and CB amplification were under the minimum level of detection, being considered as undetermined. B) Immunoblot showing expression of full length dystrophin protein in CB from normal mice and absence in CB from *mdx* mice.

To understand the origin of the blunted HVR observed in *mdx* mice, we investigated the CB, the major chemosensory organ responsible to sense changes in PaO_2_ as well as changes in PaCO_2_ and pH. First, we used qPCR to determine that dystrophin mRNA is expressed in CB of normal mice albeit at levels less than in TA muscle, however, it is absent in *mdx* mice (Figure 5A). The delta Ct values obtained from normal TA and CB were 18. 85±0.31 and 25.51±0.07, respectively (mean ± SD). As expected, dystrophic TA and CB yielded undetermined delta CT values, indicating that dystrophin is expressed in normal CB but not in dystrophic CB. Second, we used Western blotting to determine that full length dystrophin protein is expressed in the CB of normal mice, however, it was absent in *mdx* mice (Figure 5B). These results demonstrate that dystrophin mRNA and protein is expressed in normal CB and supports the idea that its absence in dystrophic CB may be, in part, responsible for the alterations in chemosensory drive observed in *mdx* mice.

### Blunted chemosensory drive in *mdx* mice

**Figure 6:**
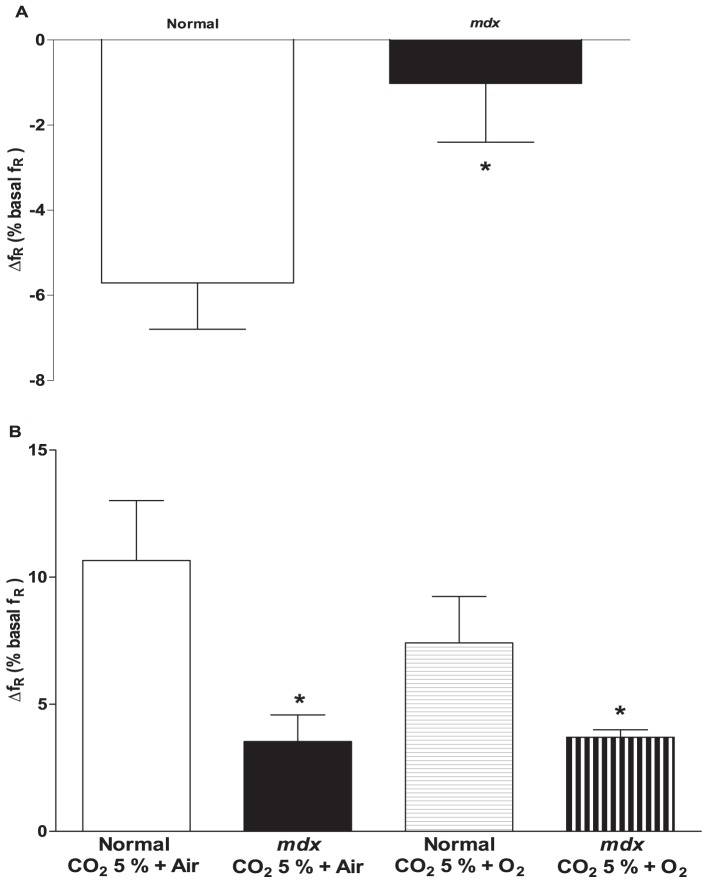
Reduced peripheral chemosensory drive in anesthetized *mdx* mice. A) The Dejours test adapted to small rodents was utilized and the first 10 s of hyperoxia exposure were analyzed. The peripheral chemosensory drive was significantly affected in *mdx* mice compared to normal mice. B) The first 10 s of exposure to air-balanced hypercapnia or O_2_-balanced hypercapnia were analyzed as the fast component of the HCVR. The HCVR induced by air-balanced hypercapnic gas mix (empty bar) was attenuated in normal mice compared to the HCVR induced by the hyperoxia-balanced hypercapnia mix (hatched bar). In the *mdx* mice, the early HCVR induced by air-balanced hypercapnic gas mix (gray bar) was similar to the early HCVR induced by the hyperoxia-balanced hypercapnia gas mix (filled bar). The analysis also showed a significant attenuation of the fast component of the HCVR in *mdx* mice compared to normal mice exposed to either air-balanced or hyperoxia-balanced hypercapnic gas mixtures. Mean ± SEM; * p<0.05, *** p<0.001. n = 4.

The results presented above allowed us to hypothesize that CB is responsible for the blunted ventilatory response in *mdx* mice. To test the hypothesis that dystrophin may play a role in the CB chemosensory drive, we performed two tests that can report the functioning of the CB chemosensory drive: the Dejours test [54] adapted to small animals [55], [56] and tests for the fast (peripheral) and slow (central) component of the HCVR to dissect the response induced by CB and the central chemosensory neurons [57], [58]. In normal anesthetized mice, exposure to hyperoxia (FiO_2_ 100%) induced a reduction of the *f*_R_ during the first 10 s of exposure demonstrating normal CB functioning; however, a positive Dejours test or significant attenuation of the hyperoxia-induced reduction *f*_R_ was observed in anesthetized *mdx* mice (Figure 6A; Table S4). As expected for the Dejours test, the V_T_ (percentage of basal) of the HVR was increased compared to normoxia in both genotypes (Figure 6B). The V_T_ from *mdx* was higher than in normal, although this difference was not statistically significant (Figure S3).

To accurately dissect the peripheral (fast response) and central (slow response) from the total HCVR, we exposed anesthetized animals to one minute of an air-balanced hypercapnic gas mixture obtaining a full peripheral chemosensory response. Animals were first allowed to recover (normoxia for 10 minutes) and then challenged using a hyperoxia-balanced hypercapnic gas to inhibit the tonic peripheral chemosensory response. The *f*_R_ of *mdx* mice was significantly reduced compared to the *f*_R_ in normal mice after a 10 s exposure to air-balanced hypercapnic or hyperoxia-balanced hypercapnic gas mixtures. This result demonstrates attenuation in the fast component of the HCVR in *mdx* mice (Figure 6B; Table S5). As expected, the normal mice reduced the *f*_R_ when exposed to a hyperoxia-balanced hypercapnic gas compared to an air-balanced hypercapnic gas, showing that hyperoxia is able to chemically inhibit the tonic activity of the CB. In contrast, we did not observe any difference in *f*_R_ of *mdx* mice for either air-balanced hypercapnic or hyperoxia-balanced hypercapnic gas mixtures. These results also suggest that the peripheral CB chemosensory drive rather than central chemosensory drive is attenuated in the 6–7 month-old *mdx* mice.

## Discussion

The present study supports the hypothesis that the absence of dystrophin impairs normal ventilation and extends it to the ventilatory response to mild hypoxia, manifesting as hypoxic desensitization or a blunted response to new hypoxic stimuli in the *mdx* mice. Moreover, our HVR findings suggest, based on Dejours test and fast component of HCVR, that this blunted HVR is mediated by the CB (the major chemosensory organ for arterial oxygen, carbon dioxide and pH concentrations). These features affect the arterial blood gas concentration and pH as well as the ability to respond to hypoxic stresses *in vivo*. The absence of dystrophin in the CB, confirmed by immunoblotting and gene expression analyses, may provide a mechanistic basis for the impaired peripheral chemosensory drive and blunted ventilatory response in *mdx* mice.

### Blunted HVR and impaired hypoxic stress test in *mdx* mice

Using a custom built WBP chamber we recorded ventilatory values that are well within the range of previous reports studying ventilatory parameters in mice [59], [60], [61], [62], [63], [64]. Consistent with most [44], [45], [47], [50], [59], [60], [61], [62], [63], [64] but not all reports [48], [49], *mdx* mice showed a significantly lower *f*_R_, reduced V_T_ and 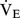 during normoxia compared to normal mice. (Fig. 1 and Table S1). *Mdx* mice showed impairment of HVR when challenged with mild hypoxic challenges (FiO_2_ 18 and 15%), however, but not during moderate (FiO_2_ 10 and 12%) and severe hypoxic challenges (FiO_2_ 8%; Figure 1B). As expected for a small animal like the mouse, tachypnea rather than increasing V_T_ was the means utilized to achieve net changes in 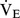 while responding to hypoxia.

The normoxic *f*_R_ range reported in normal mice, independently of the strain, age and method of measurement (direct plethysmograph, pneumotachograph and WBP) varies between 2.8 and 5 Hz (167 and 300 breaths per minute) [59], [60], [61], [63], [65], [66]. Our measurements were based on the guidelines for standard pre-clinical experiments in *mdx* mouse to minimize stress, behavioral and physical activity that might influence in the ventilatory measurements (see material & methods). The *f*_R_ obtained in this study fit in this range for both normal and *mdx* mice (3.28±0.05 *vs.* 2.95±0.06 Hz, respectively). Age related changes in these parameters have shown by Dupont-Versteegden et al.; 3 month-old *mdx* mice have a higher *f*_R_ compared to 1 year-old *mdx* mice and that 1 year-old *mdx* mice have lower *f*_R_ than normal mice [44], [45]. In contrast, Ishizaki et al compared 2 and 4 months-old normal to age matched *mdx* mice and showed a significant decrease in *f*_R_ and in 7 month-old *mdx* mice a significant increase in *f*_R_ and a significant decrease in V_T_ [49]. Huang et al. showed a significant decrease in the *f*_R_ and in V_T_ at 3 and 6 month-old *mdx* mice [48]. However, the normoxic *f*_R_ reported for normal and *mdx* mice by both Ishizaki et al (2008) and Huang et al (2011) studies are extremely elevated (c. 6 Hz or 400 breaths per minute) compared to previous reports from the most common inbred strains [59], [60], [62], [63], [64], [65]. However, in agreement with our results and within the *f*_R_ range reported for normal mice as discussed above [59], [60], [61], [63], [65], [66], Dupont-Versteegden et al. have shown using a similar WBP chamber used by Ishizaki et al. and Huang et al. and with a similar procedure described on the published guidelines for standard pre-clinical experiments in *mdx* mouse [67] and used by us that 1 year-old *mdx* mice have lower *f*_R_ than normal mice [44], [45].

To determine whether the blunted HVR was due to abnormal phrenic nerve discharge, we studied *in situ* phrenic nerve activity. The phrenic nerve discharge analysis demonstrated a significant reduction of *f*_x_ in *mdx* mice during normoxia (FiO_2_ 21%) and at FiO_2_ 100% compared to normal mice, independently confirming the lower *f*_R_ observed in the WBP experiments and suggesting abnormalities in the CB chemosensory response. However, our recordings were not designed to interrogate diaphragmatic function. The burst frequency analysis showed no difference between the genotypes indicating that the phrenic nerve's motor neurons activity is normal. Personius and co-workers previously showed that *in vitro* stimulation of the *mdx* mice phrenic nerve at 35 Hz (to simulate physiological firing frequency), showed an augmentation of neural transmission variability with reduced ability to precisely and consistently generate the same muscle force and a greater contractile fatigue in dystrophic diaphragm following both nerve and muscle stimulation [43]. Although the cause of reduced performance of the phrenic nerve of *mdx* mice is unclear, the results along with diaphragmatic muscle damage [39] would explain, in part, the lower 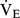
during normoxic conditions observed here.

We observed an impaired ability to respond abrupt change from normoxia to severe hypoxic challenges (PO_2_ 8%) in *mdx* mice compared to normal mice. Previously, it has been demonstrated that mice with different genetic backgrounds have different HVR patterns and resistance to hypoxia [59], [63], [65], and those that display a blunted HVR response also have a weaker compensation in response to severe hypoxia [65]. Interestingly, we have previously reported that dystrophic *Drosophila* also has an impaired capacity to respond to hypoxic stress [68]. Our ventilatory data from *mdx* mice (bradypneic, blunted HVR during normoxia and lower resistance to severe hypoxia challenges) would also support previous data showing a severe functional deficit of the damaged diaphragm. This is of significance as the progression of the kyphosis would be predicted to further impair pulmonary function in *mdx* mice [39], [69], as it does in DMD patients [70], [71], [72].

### Hypoxemia, hypercapnia and reduced CB chemosensory drive

*Mdx* mice presented a blunted response to mild hypoxia and bradypenia during normoxic conditions compared to age-matched control mice. As expected, the major consequence of the chronic hypoventilation was altered blood gases, including hypoxemia, hypercapnia and acidosis. Our ventilatory and blood gas results are in line with previous data from *mdx* mice [44], [45] and resemble hypoventilated DMD patients with hypoxemia and hypercapnia [3], [5], [73], [74] and acidosis [75], [76]. As expected, we observed a significant increase in hematocrit in *mdx* mice, possibly due to hypoxic condition. Chronic hypoxia is known to induce erythropoiesis [77], [78], [79]. Our hematocrit values were in the range of values for hematocrit obtained from normal and *mdx* mice reported by others [44], [45], [78].

It is generally accepted that in mammals the CB play a central role in the regulation of ventilation and its activity is modulated by changes in the PaO_2_, PaCO_2_ and pH. The functional unit of chemoreception is called glomoid, composed by the chemoreceptors glomic cells (Type I cells), which is surrounded by sustentacular cells (Type II) cells and is innervated by sensory neurons of the carotid sinus nerve (CSN). The CSN, which somas are located in the petrosal ganglion, innervates the caudal area of the NTS, which then connects to the respiratory groups of the brain stem. Changes in these arterial parameters of PaO_2_, PaCO_2_ and pH depolarizes the membrane of glomic cells, increasing cytosolic [Ca^2+^], which results into release of excitatory neurotransmitters, increasing the activity of carotid sinus nerve. The increase of CB's activity and thus the firing activity of CSN results in an adaptive ventilatory response, such as HVR and HCVR [32], [33], [80]. Denervation or excision of CBs reduces or abolishes the HVR in rats, cats, dogs, goats and humans [81], [82], [83], [84], [85], [86], [87], [88]. In mice, the HVR is abolished after CSN denervation, showing that hypoxia-induced 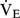 increase (due to decrease of *f*_R_ and no change in the V_T_) is mediated by CB [89]. CB denervation also causes attenuation of the HCVR; however, this attenuation is not observed in all species and it is chronically compensate by central chemoreceptors [88], [90], [91]. In this context, it has been shown that the CB is responsible for a greater-than-additive ventilatory response in awake and anesthetized animals when hypoxic and hypercapnic stimuli are given together, compared to only one stimulus separately [92], [93], [94], [95]. In mice, the CB can also drive phrenic nerve activity via the NTS and respiratory neurons [32], [33]. Chemo- or electrical stimulation of CSN increases phrenic nerve activity and thus ventilation [96], [97]. Bilateral denervation of the CB abolishes phrenic nerve activity induced by chemo- or electrical stimulation [96], [97]. One of the major consequences of the chronic hypoxemic condition is the desensitization response of the CB to hypoxic stimulus. This response is characterized by a blunted HVR to new hypoxic challenges and has been described in humans and animal models exposed to chronic hypoxia [98], [99]. However, the role of dystrophin in the chemosensory response of the CB and the control of ventilation are unknown. Our ventilatory results together with the absence of gene and protein expression in *mdx* CB suggest that the absence of dystrophin in the CB may decrease the peripheral chemosensory sensitivity in *mdx* mice. Since the CB contains numerous cell types/structures including Type I and Type II cells, smooth muscle cells, ganglion cells and nerve endings that are all capable of expressing dystrophin it is currently unclear which cellular compartment(s) are responsible for the decreased chemosensory sensitivity we noted in *mdx* mice.

We performed the Dejours test to investigate the chemosensory drive of the CB chemoreceptors. Our results showed a significant difference in hyperoxia-induced attenuation of the *f*_R_, suggesting an impairment of the chemosensory drive and thus an impairment of the CB response to different levels of oxygen (Fig. 6). This idea is also supported by our HVR experiment (Figure 1), in which *mdx* mice are unable to adapt their *f*_R_ to a wide range of FiO_2_ (from 15 to 100%). We also showed in both awake and anesthetized animals that *mdx* mice presented similar responses as normal mice at mild and more severe hypoxic stimuli (from FiO_2_ 12 to 0%), suggesting that the HVR is affected only at physiological ranges of hypoxia.

### HCVR and contribution of the CB to the fast component of HCVR

Consistent with previous studies, we found no impairment of overall (i.e. when considering both the fast and slow components) HCVR in 6–7 month old *mdx* mice compared to age-matched controls [47], [50]. In general, both *mdx* and normal mice presented a steady and robust increase in *f*_R_ and V_T_ after 60 seconds of exposure to all hypercapnic challenges. Only at 16 months-old *mdx* mice show a significant HCVR increase compared to normal mice [47], [50]. Our results support the previously published HCVR results, where there is no difference between the two genotypes at 6–7 months-old. Studies show that, the HCVR occurs between 5–10 s after exposure to hypercapnia challenges and this response is delayed in CB chemodenervated dogs [54], [100], [101]. Data from CB-denervated human [57], [102] and animals [58], [101], [103] demonstrates that HCVR is mainly controlled by the central chemosensory neurons rather than CBs. However the CBs are responsible for 20–50% of the total HCVR [103], [104], [105], [106], [107], and are singularly responsible for the first seconds (fast component) of the HCVR [57], [101], [108]. Consistent with this, we observed a significant attenuation of the *f*_R_ in the HCVR of *mdx* mice compared to normal mice during the first 10 s of exposure to two protocols of hypercapnia (balanced in air or in oxygen), suggesting that CB chemosensitivity is attenuated due to dystrophin deficiency in mdx mice and the intact HCVR response after 60 s of hypercapnic stimulus suggests that the central chemoreceptor neurons are responsible for the late component of the HCVR observed in Figure 2.

### Relevance to DMD and Conclusions

Increasing our understanding of the mechanisms of respiratory failure is extremely important in the appropriate management and development of therapeutics for DMD. The lifespan of DMD patients is dramatically reduced once the VC falls below 1L [5]. The progressive decrease in VC is correlated with age and hypoxemia [5], [73], [74], [109]. Since hypoxia is known to cause impaired muscle function and muscle damage [110], [111], [112], [113], chronic hypoxia would be predicted to further aggravate the disease process. Indeed, 6 month-old *mdx* mice exposed to 8 h/day episodic hypoxia (PO_2_ 5%) for 12 weeks showed impaired diaphragmatic contraction relative to aged matched normoxic *mdx* mice [46]. While we are unaware of previous investigations of chemosensory respiratory drive integrity in DMD *per se*, DMD patients are known to suffer from hypoventilation and apneic episodes during sleep and decreased arterial oxygen saturation [109], [114] which may in part, be due to diminished CB-mediated drive mechanisms.

To facilitate our understanding of dystrophic patho-physiology as well as assist in therapeutic development, in this study we investigated respiratory control mechanisms in the *mdx* mouse model of DMD. *Mdx* mice had significant respiratory and systemic abnormalities as revealed by their respiratory parameters, HVR and blood gas analyses. The lack of dystrophin in *mdx* CB was functional, since *mdx* mice had a defective peripheral chemosensory drive that would be predicted to contribute, alongside the well documented diaphragmatic pathology, to the respiratory failure. Given the evolutionary conservation of the dystrophin gene as well as many components of the hypoxia sensing and respiratory functioning, we suggest that CB functioning and peripheral chemosensory drive mechanisms should be investigated in DMD patients as they may be compromised and indeed contribute to respiratory failure.

## Supporting Information

Figure S1 **Photography of custom made WBP chamber and Nose Plethysmograph.** A. WBP with 150 ml volume. Inside the WBP chamber, a flat white surface to give stability to the mouse. B. The custom made Nose Plethysmograph from a 15 ml conical tube.
(PDF)

Figure S2
**Hypercapnic ventilatory response obtained from normal and** ***mdx*** **mice.** WBP from normal (empty bars) and *mdx* (filled bars) exposed to different levels of hypercapnia: FiCO_2_ 5 and 10% mixed with air or O_2_.A. Respiratory rate (*f*_R_, Hz). B. Tidal volume normalized to body weight (V_T_, µL/g). C. Normalized minute ventilation (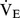, µL/s/g). Mean ± SEM; * p<0.05; ** p<0.01, n = 5.(PDF)

Figure S3 **Percentage of basal changed V_T_ during Dejour's Test.** Dejour's test V_T_ obtained from normal (empty bars) and *mdx* (filled bars) was normalized to the V_T_ obtained during normoxia. The difference did not reach statistical significance. Mean ± SEM; n = 5.
(PDF)

Table S1 **HVR for normal and** ***mdx*** **mice exposed to different FiO_2_.**
(PDF)

Table S2 **Phrenic nerve discharge from normal and** ***mdx*** **mice challenged to different FiO_2_ exposure.**
(PDF)

Table S3 **HCVR for normal and** ***mdx*** **mice challenged to different FiCO_2_ exposure.**
(PDF)

Table S4 **Chemosensory drive in** ***mdx*** **by Dejours maneuver.** Values were analyzed 10 s after being exposed to hyperoxia and normalized to normoxic baseline.
(PDF)

Table S5 **Early HCVR in** ***mdx*** **mice.** Values were analyzed 10 s after being exposed to hyperoxia and normalized to normoxic baseline.
(PDF)
