# Supplementary figures and images for: Ventilatory Chemosensory Drive Is Blunted in the mdx Mouse Model of Duchenne Muscular Dystrophy (DMD)
Source: PLoS One. 2013 Jul 29;8(7):e69567. doi: 10.1371/journal.pone.0069567 (PMC3726676; doi:10.1371/journal.pone.0069567)

A

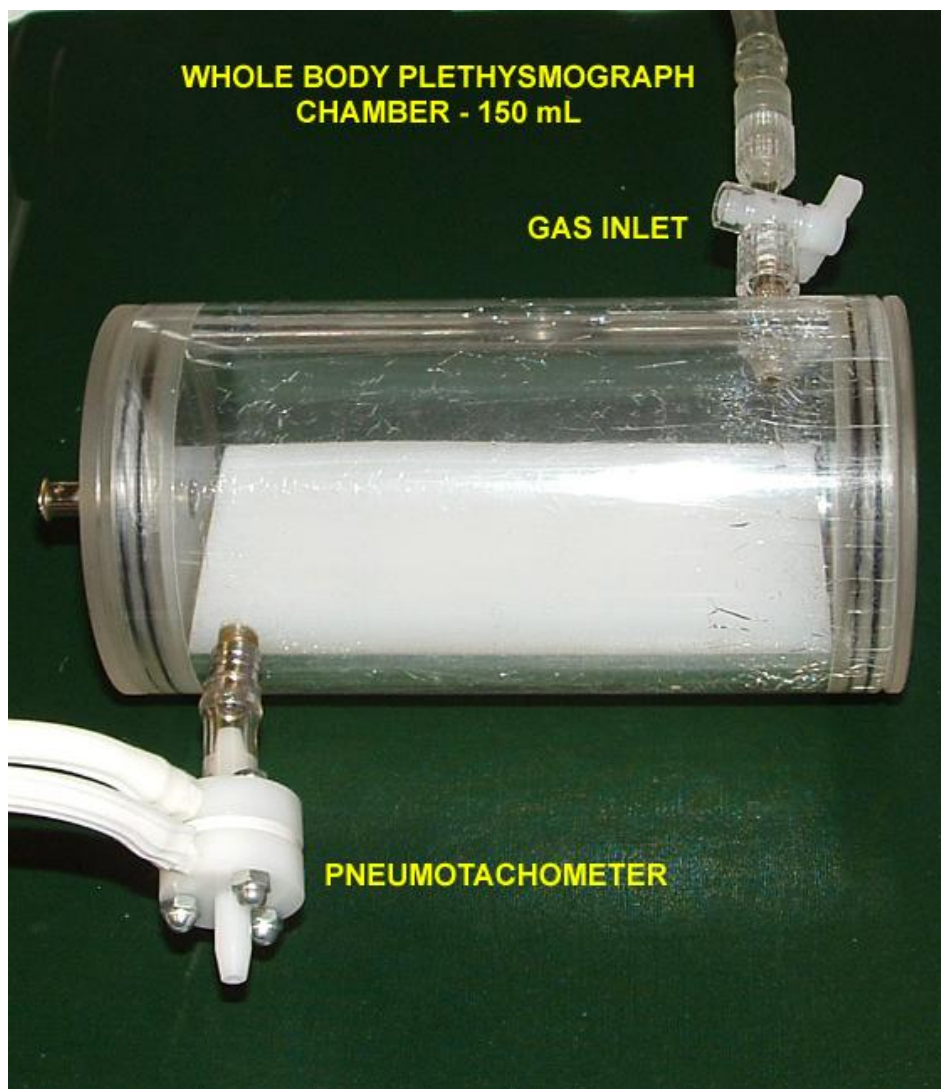

B

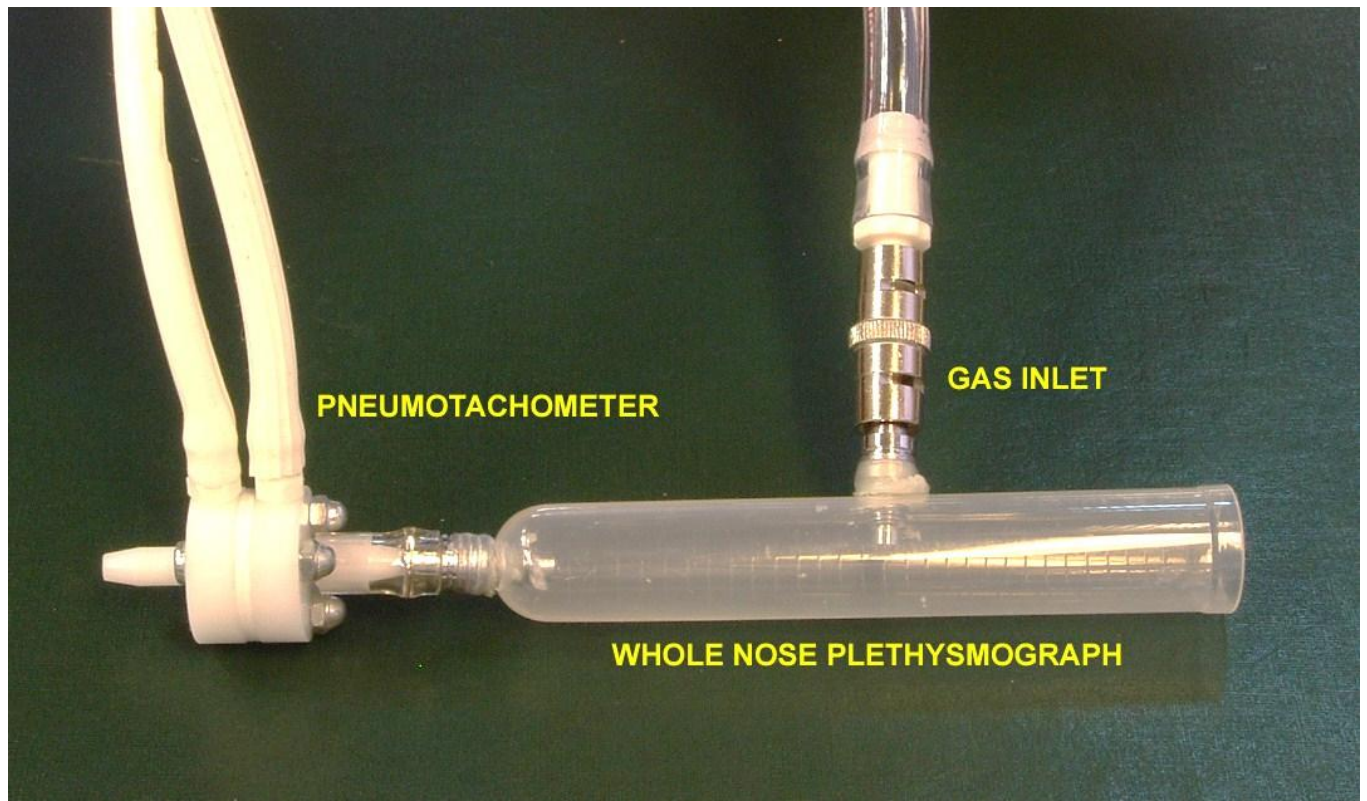

Supplement: Figure S1 — Photography of custom made WBP chamber and Nose Plethysmograph. A. WBP with 150 ml volume. Inside the WBP chamber, a flat white surface to give stability to the mouse. B. The custom made Nose Plethysmograph from a 15 ml conical tube. (PDF) [file pone.0069567.s001.pdf]

**a**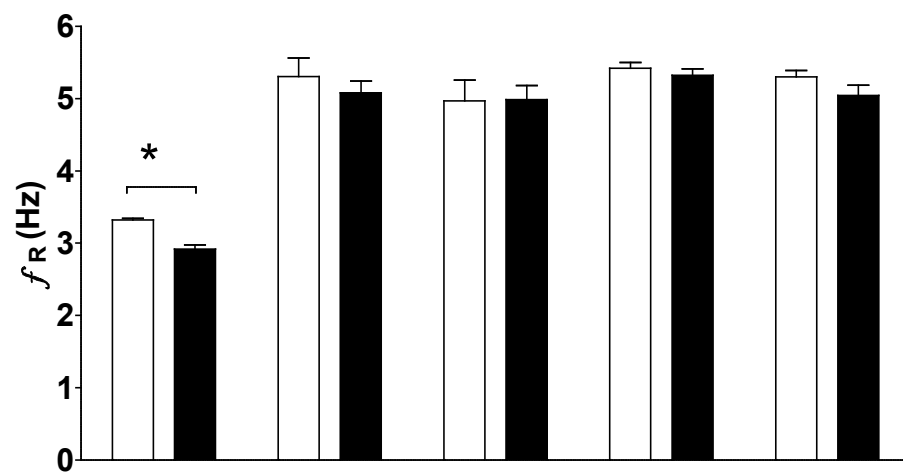**b**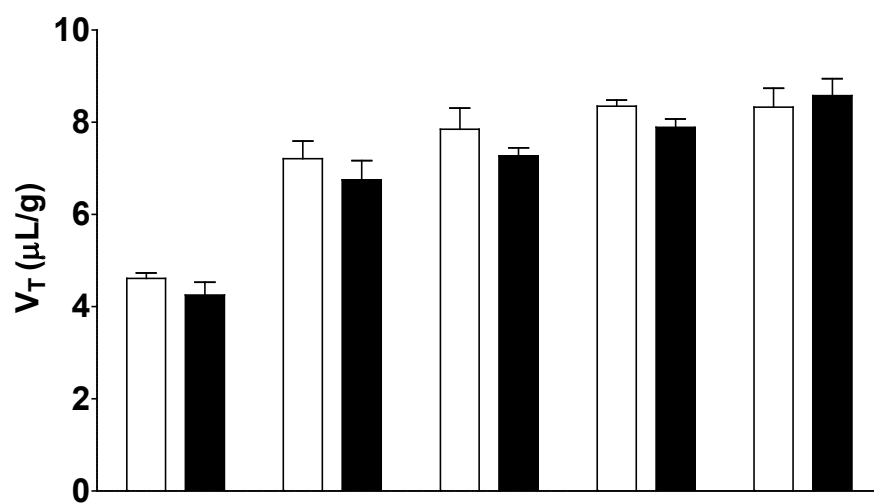**c**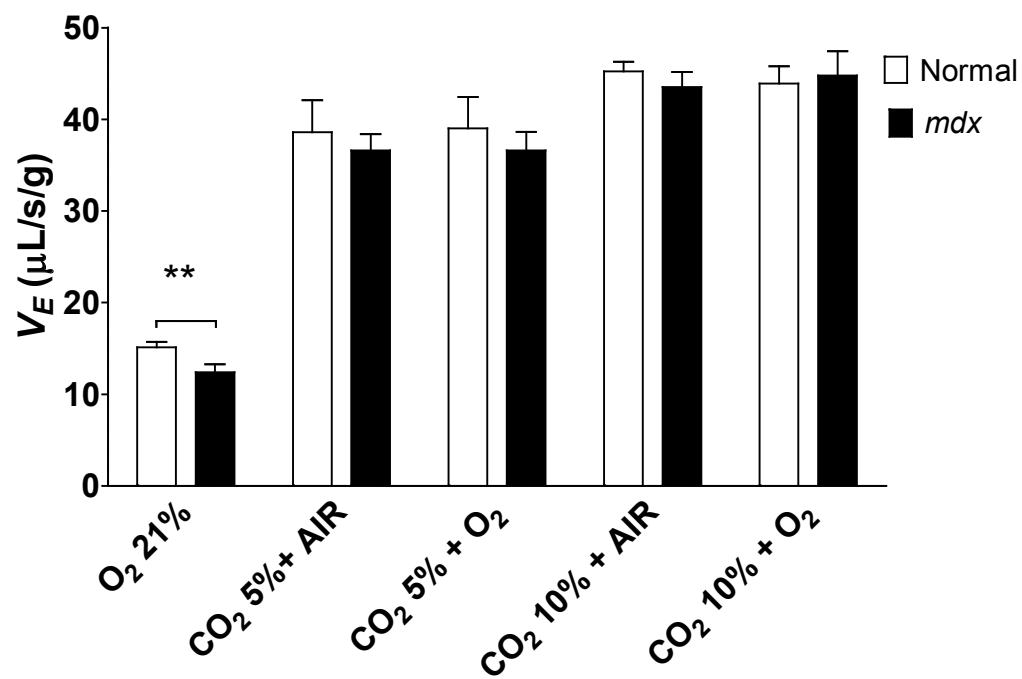

Supplement: Figure S2 — Hypercapnic ventilatory response obtained from normal and mdx mice. WBP from normal (empty bars) and mdx (filled bars) exposed to different levels of hypercapnia: FiCO2 5 and 10% mixed with air or O2.A. Respiratory rate (f R, Hz). B. Tidal volume normalized to body weight (VT, µL/g). C. Normalized minute ventilation (, µL/s/g). Mean ± SEM; * p<0.05; ** p<0.01, n = 5. (PDF) [file pone.0069567.s002.pdf]

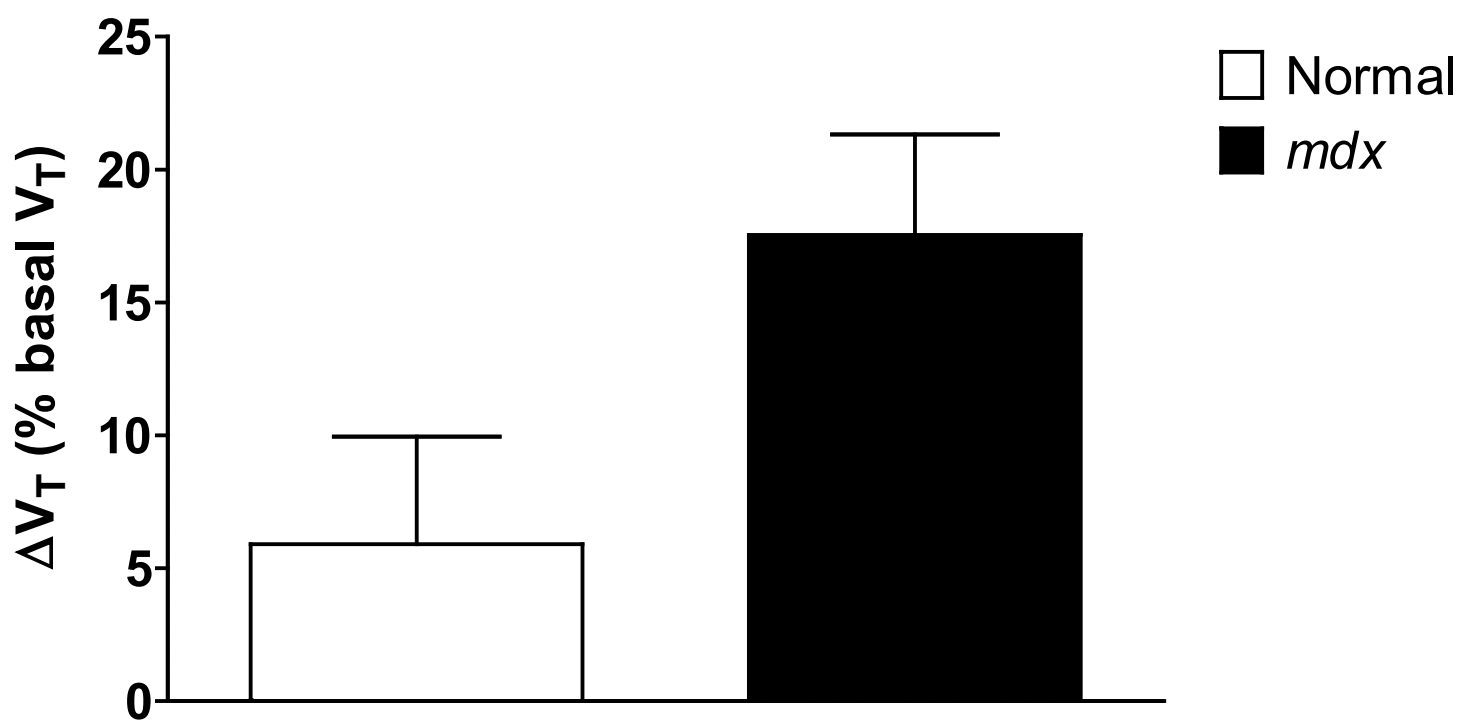

Supplement: Figure S3 — Percentage of basal changed VT during Dejour's Test. Dejour's test VT obtained from normal (empty bars) and mdx (filled bars) was normalized to the VT obtained during normoxia. The difference did not reach statistical significance. Mean ± SEM; n = 5. (PDF) [file pone.0069567.s003.pdf]
