# Supplementary material for: Ventilatory Chemosensory Drive Is Blunted in the mdx Mouse Model of Duchenne Muscular Dystrophy (DMD)
Source: PLoS One. 2013 Jul 29;8(7):e69567. doi: 10.1371/journal.pone.0069567 (PMC3726676; doi:10.1371/journal.pone.0069567)
Supplement: Table S1 — HVR for normal and mdx mice exposed to different FiO2. (PDF) [file pone.0069567.s004.pdf]

Table S1. HVR for normal and *mdx* mice exposed to different FiO_2.

**FiO_2 (%)**		**21**	**18**	**15**	**12**	**10**	**8**	**100**
**f_R** (Hz)	Normal	3.28 ± 0.05	3.36 ± 0.7	3.70 ± 0.09	4.20 ± 0.08	4.28 ± 0.30	5.12 ± 0.20	2.90 ± 0.10
	mdx	2.95 ± 0.06*	3.05 ± 0.12*	3.22 ± 0.07*	4.00 ± 0.08	4.37 ± 0.060	5.10 ± 0.21	2.80 ± 0.04
**V_T** (µl/g)	Normal	4.97 ± 0.29	4.16 ± 0.08	4.57 ± 0.17	4.92 ± 0.15	4.85 ± 0.13	5.19 ± 0.28	4.02 ± 0.14
	mdx	4.39 ± 0.08*	4.23 ± 0.06	4.52 ± 0.09	5.20 ± 0.07	5.11 ± 0.10	4.94 ± 0.12	4.66 ± 0.21*
**V_E** (µl/s/g)	Normal	16.75 ± 0.99	15.74 ± 0.39	17.84 ± 1.18	19.57 ± 1.00	20.72 ± 0.97	26.59 ± 2.23	12.39 ± 0.53
	mdx	13.14 ± 0.33*	12.77 ± 0.27*	14.81 ± 0.35*	21.72 ± 0.53	21.05 ± 0.62	24.33 ± 1.70	14.79 ± 0.54

Values are expressed as means ± SEM (n=5). * $P < 0.05$.
