# Supplementary material for: Ventilatory Chemosensory Drive Is Blunted in the mdx Mouse Model of Duchenne Muscular Dystrophy (DMD)
Source: PLoS One. 2013 Jul 29;8(7):e69567. doi: 10.1371/journal.pone.0069567 (PMC3726676; doi:10.1371/journal.pone.0069567)
Supplement: Table S2 — Phrenic nerve discharge from normal and mdx mice challenged to different FiO2 exposure. (PDF) [file pone.0069567.s005.pdf]

Table S2. Phrenic nerve discharge from normal and *mdx* mice challenged to different FiO_2 exposure.

**FiO_2 (%)**		**21**	**8**	**4**	**0**	**100**
f_x (Hz)	Normal	3.10 ± 0.19	3.78 ± 0.24	3.97 ± 0.28	4.80 ± 0.36	2.43 ± 0.14
	mdx	2.30 ± 0.11*	3.58 ± 0.06	3.88 ± 0.10	4.05 ± 0.14	1.83 ± 0.13*

Values are expressed as means ± SEM (n=4). * $P< 0.05$.
