# Supplementary material for: Ventilatory Chemosensory Drive Is Blunted in the mdx Mouse Model of Duchenne Muscular Dystrophy (DMD)
Source: PLoS One. 2013 Jul 29;8(7):e69567. doi: 10.1371/journal.pone.0069567 (PMC3726676; doi:10.1371/journal.pone.0069567)
Supplement: Table S3 — HCVR for normal and mdx mice challenged to different FiCO2 exposure. (PDF) [file pone.0069567.s006.pdf]

Table S3. HCVR for normal and *mdx* mice challenged to different $FiCO_2$ exposure.

$FiCO_2$ (%)		5% CO_2 + 95%AIR	5% CO_2 + 95% O_2	10% CO_2 + 90% AIR	10% CO_2 + 90% O_2
f_R (Hz)	Normal	5.3 ± 0.26	4.97 ± 0.29	5.42 ± 0.08	5.3 ± 0.09
	mdx	5.08 ± 0.16	4.99 ± 0.20	5.32 ± 0.09	5.04 ± 0.14
V_T (µl/g)	Normal	7.21 ± 0.38	7.85 ± 0.45	8.35 ± 0.13	8.32 ± 0.40
	mdx	6.75 ± 0.41	7.27 ± 0.17	7.89 ± 0.18	8.58 ± 0.37
V_E (µl/s/g)	Normal	38.58 ± 3.49	39.00 ± 4.42	45.21 ± 1.07	43.90 ± 1.88
	mdx	36.59 ± 1.78	36.60 ± 2.02	43. 54± 1.63	44.80 ± 2.63

Values are expressed as means ± SEM (n=5). * P< 0.05.
