# Supplementary material for: Ventilatory Chemosensory Drive Is Blunted in the mdx Mouse Model of Duchenne Muscular Dystrophy (DMD)
Source: PLoS One. 2013 Jul 29;8(7):e69567. doi: 10.1371/journal.pone.0069567 (PMC3726676; doi:10.1371/journal.pone.0069567)
Supplement: Table S4 — Chemosensory drive in mdx by Dejours maneuver. Values were analyzed 10 s after being exposed to hyperoxia and normalized to normoxic baseline. (PDF) [file pone.0069567.s007.pdf]

Table S4. Chemosensory drive in *mdx* by Dejours maneuver. Values were analyzed 10 s after being exposed to hyperoxia and normalized to normoxic baseline.

| | $\boldsymbol{f_R}$ (%) | $\mathbf{V_T}$ (%) | $\mathbf{V_E}$ (%) |
|---|---|---|---|
| Normal | -5.71 ± 1.09 | 5.90 ± 4.06 | 0.32 ± 3.55 |
| *mdx* | -1.02 ± 1.38* | 17.54 ± 3.79 | -13.50 ± 3.96* |

Values are expressed as means ± SEM (n=5). * P< 0.05.
