# Supplementary material for: Ventilatory Chemosensory Drive Is Blunted in the mdx Mouse Model of Duchenne Muscular Dystrophy (DMD)
Source: PLoS One. 2013 Jul 29;8(7):e69567. doi: 10.1371/journal.pone.0069567 (PMC3726676; doi:10.1371/journal.pone.0069567)
Supplement: Table S5 — Early HCVR in mdx mice. Values were analyzed 10 s after being exposed to hyperoxia and normalized to normoxic baseline. (PDF) [file pone.0069567.s008.pdf]

Table S5. Early HCVR in *mdx* mice. Values were analyzed 10 s after being exposed to hyperoxia and normalized to normoxic baseline.

**FiCO$_2$ (%)**		**5% CO$_2$ + 95% AIR**	**5% CO$_2$ + 95% O$_2$**
f_R (%)	Normal	10.66 ± 2.36	7.41 ± 1.83
	mdx	3.53 ± 1.05*	3.71 ± 0.28*
V_T (%)	Normal	29.48 ± 7.77	12.60 ± 5.01
	mdx	12.89 ± 3.14*	3.32 ± 0.39
V_E (%)	Normal	46.09 ± 10.35	20.68 ± 5.15
	mdx	17.29 ± 4.27*	7.33 ± 0.54

Values are expressed as means ± SEM (n=5). * P< 0.05.
